# Opioid-Sparing Effects of Perioperative Ketamine With Inconsistent Analgesic Benefit in Orthopedic Surgery: A Systematic Review of Controlled Trials

**DOI:** 10.7759/cureus.112926

**Published:** 2026-07-18

**Authors:** Mackenzie Buckholz, Armon Moradian, Rohan M Patel, Ashley Forrest, Olivia Pisarski, Peter Cohen, Robin J Jacobs

**Affiliations:** 1 Department of Medicine, Nova Southeastern University Dr. Kiran C. Patel College of Osteopathic Medicine, Fort Lauderdale, USA

**Keywords:** multimodal analgesia, opioid-sparing, orthopedic surgery, perioperative ketamine, postoperative pain, randomized controlled trials

## Abstract

Effective postoperative pain management following orthopedic surgery remains a challenge, particularly in the context of increasing opioid-related morbidity. Multimodal analgesic strategies incorporate adjuvant therapies such as ketamine to reduce opioid requirements while maintaining adequate pain control. This systematic review evaluated the efficacy and safety of perioperative ketamine as an adjunct to opioid therapy for acute postoperative pain management in adults undergoing orthopedic procedures. A PRISMA-guided search of Ovid MEDLINE, Embase, Web of Science, and Cochrane CENTRAL identified randomized controlled trials published between January 2014 and September 2024. Eligible studies included patients aged 18-65 years undergoing orthopedic surgery who received perioperative ketamine compared to opioid-based regimens. Primary outcomes included postoperative opioid consumption and patient-reported pain scores, while secondary outcomes included adverse events. Risk of bias was assessed using the Joanna Briggs Institute (JBI) Critical Appraisal Checklist for Randomized Controlled Trials. Due to heterogeneity in ketamine dosing, administration, and outcome reporting, a qualitative synthesis was performed. Eleven randomized controlled trials met the inclusion criteria. Adjunctive ketamine reduced postoperative opioid consumption in five of 11 studies, with the most consistent effects observed within the first 24 hours postoperatively. Four studies reported statistically significant improvements in patient-reported pain scores, while others demonstrated no significant differences despite reduced opioid use. Ketamine showed a safety profile comparable to opioid-only regimens, with no consistent increase in adverse events; several studies reported reductions in opioid-related side effects such as pruritus and sedation. Perioperative ketamine may reduce postoperative opioid consumption without increasing adverse events, although its effect on pain intensity remains variable. These findings support its use as part of multimodal analgesia strategies aimed at minimizing opioid exposure, though further research is needed to standardize dosing protocols and evaluate long-term outcomes.

## Introduction and background

Orthopedic surgical procedures are increasingly common in the United States owing to an aging population, the rising prevalence of obesity, and continued advancements in surgical techniques [1,2]. Musculoskeletal conditions account for a substantial proportion of healthcare utilization, representing a leading cause of outpatient visits nationwide [3]. Among these conditions, osteoarthritis and other degenerative joint diseases contribute significantly to surgical demand and associated healthcare burden.

Total hip and knee arthroplasty are well-established and effective interventions for patients with advanced osteoarthritis who have failed conservative management [4]. According to the Healthcare Cost and Utilization Project (HCUP), orthopedic procedures - including knee and hip arthroplasty, spinal fusion, femur fixation, and vertebral discectomy - rank among the most frequently performed inpatient surgeries in the United States [5]. These procedures require a comprehensive perioperative approach, with effective postoperative pain management playing a critical role in optimizing recovery and functional outcomes.

Opioids remain a cornerstone of postoperative analgesia for moderate to severe pain. These agents exert their effects primarily through μ-opioid receptor agonism, inhibiting nociceptive transmission at both spinal and supraspinal levels [6]. Although effective, opioid use is associated with well-documented adverse effects, including central nervous system depression, respiratory depression, nausea, vomiting, constipation, pruritus, and potential endocrine dysfunction [6]. Commonly used perioperative opioids include fentanyl, morphine, hydromorphone, oxycodone, and tramadol, selected based on pharmacokinetic properties, potency, and route of administration [7]. In response to growing concerns regarding opioid-related morbidity, dependence, and misuse, perioperative pain management strategies have shifted toward opioid-sparing, multimodal approaches. Current multidisciplinary guidelines emphasize minimizing opioid exposure while maintaining adequate analgesia through the use of non-opioid adjuncts [8].

Ketamine, an N-methyl-D-aspartate (NMDA) receptor antagonist, possesses both anesthetic and analgesic properties and has emerged as a potential adjunct in multimodal analgesia regimens [9]. In addition, it exhibits anti-hyperalgesic properties by attenuating central sensitization and reducing nociceptive amplification, thereby helping prevent postoperative pain hypersensitivity while decreasing opioid requirements. Prior studies have demonstrated reductions in postoperative opioid consumption and, in some cases, improvements in pain scores among orthopedic surgical populations receiving perioperative ketamine [10]. Additionally, ketamine has been associated with a reduction in opioid-related adverse events, such as postoperative nausea and vomiting, compared to opioid monotherapy [11].

Effective control of acute postoperative pain is essential for facilitating early mobilization, improving patient satisfaction, and enhancing recovery trajectories following orthopedic surgery [12-14]. Multimodal analgesia strategies combining opioids with adjuvant agents have, therefore, become standard practice, replacing traditional opioid-centric approaches [8,12-14]. These principles are consistent with Enhanced Recovery After Surgery (ERAS) protocols, which emphasize multimodal, opioid-sparing analgesia to facilitate early mobilization and reduce opioid-related complications following orthopedic procedures. Within ERAS pathways, ketamine is recognized as a valuable adjunct because of its opioid-sparing properties and its ability to support effective multimodal analgesia in appropriately selected patients. Accordingly, ketamine represents a promising adjunct that may reduce opioid exposure, improve functional recovery, and mitigate adverse effects associated with higher opioid doses. However, despite increasing clinical adoption, uncertainty remains regarding the consistency of its analgesic benefit and overall safety profile in orthopedic surgical populations.

The ongoing opioid epidemic further underscores the importance of optimizing perioperative prescribing practices. Exposure to opioids in the postoperative setting has been identified as a potential pathway to long-term use and, in some cases, transition to misuse or illicit opioid consumption [15,16]. Consequently, identifying effective opioid-sparing strategies is a critical priority in perioperative care.

Previous systematic reviews and meta-analyses have reported that perioperative ketamine may reduce postoperative opioid consumption, although its effects on pain intensity have been inconsistent [10,11]. However, many earlier reviews included heterogeneous surgical populations or studies published before more recent randomized controlled trials became available [10,11]. The present review provides an updated synthesis focused specifically on contemporary controlled trials evaluating perioperative ketamine in adult orthopedic surgical populations.

This systematic review aims to evaluate the efficacy and safety of perioperative ketamine as an adjunct to opioid therapy for acute postoperative pain management in adults undergoing orthopedic surgery. It is hypothesized that ketamine use will be associated with reduced opioid consumption, improved pain control, and a lower incidence of adverse events compared to opioid monotherapy.

## Review

Methods

The objective of this review was to evaluate the efficacy and safety profile of perioperative ketamine as an adjunct to traditional opioid monotherapy for acute postoperative pain management in patients aged 18-65 years undergoing orthopedic procedures.

Protocol and Registration

This systematic review was conducted in accordance with PRISMA guidelines [17]. The review was not prospectively registered (e.g., PROSPERO).

Eligibility Criteria

Eligible studies included randomized controlled trials, controlled clinical trials, and case-controlled trials evaluating perioperative ketamine administered either alone or as an adjunct to opioids for postoperative analgesia in adults aged 18-65 years undergoing orthopedic or spinal procedures. The age restriction was applied to reduce clinical heterogeneity because older adults often have substantially different perioperative pharmacokinetics, comorbidity profiles, and analgesic requirements, which may influence both analgesic efficacy and adverse event profiles. Consequently, the findings of this review may not be generalizable to elderly orthopedic populations. Studies published between January 2014 and September 2024 were considered. Interventions included ketamine administered via any route and at any perioperative time point. These were compared against opioid-based analgesic regimens without ketamine.

Studies were excluded if they evaluated non-standard adjunctive interventions (e.g., experimental behavioral therapies or non-pharmacologic modalities) that could confound analgesic outcomes. Standard-of-care measures such as NSAIDs, acetaminophen, and early ambulation were permitted. Outcomes of interest included postoperative opioid consumption and patient-reported pain scores such as the Visual Analog Scale (VAS), the Numeric Rating Scale (NRS), and adverse events [18,19]. Studies reporting outcomes beyond three months postoperatively were excluded. Only English-language publications were included.

Search Strategy

A comprehensive literature search was conducted in Embase, Ovid MEDLINE, Web of Science, and the Cochrane Central Register of Controlled Trials (CENTRAL). The databases were last searched on September 29, 2024. Grey literature sources were not searched.

The full search strategy was adapted for each database using a combination of MeSH terms and keywords. Tables 1-4 report the Embase, Ovid Medline, Web of Science, and Cochrane Central Register of Controlled Trials (CENTRAL) search strategies.

**Table 1:** Embase search strategy. Embase: Excerpta Medica dataBASE.

Search number	Query	Results
1	'postoperative analgesia'/exp	5,141
2	'postoperative analgesi*':ab,ti,kw OR 'post-operative analgesi*':ab,ti,kw OR 'post-surg* analgesi*':ab,ti,kw OR 'postsurg* analgesi*':ab,ti,kw OR 'postoperative pain management':ab,ti,kw OR 'post-operative pain management':ab,ti,kw OR 'post-surg* pain management':ab,ti,kw OR 'postsurg* pain management':ab,ti,kw	4,715
3	#1 OR #2	6,197
4	'orthopedic surgery'/exp	16,056
5	'orthopedic surger*':ab,ti,kw OR 'orthopedic operation*':ab,ti,kw OR 'orthopedic procedure*':ab,ti,kw OR 'orthopaedic surger*':ab,ti,kw OR 'orthopaedic operation*':ab,ti,kw OR 'orthopaedic procedure*':ab,ti,kw OR 'foot surger*':ab,ti,kw OR 'foot operation*':ab,ti,kw OR 'foot procedure*':ab,ti,kw OR 'podiatric surger*':ab,ti,kw OR 'podiatric operation*':ab,ti,kw OR 'podiatric procedure*':ab,ti,kw OR 'hand surger*':ab,ti,kw OR 'hand operation*':ab,ti,kw OR 'hand procedure*':ab,ti,kw OR 'joint surger*':ab,ti,kw OR 'joint operation*':ab,ti,kw OR 'joint procedure*':ab,ti,kw OR 'joint repair*':ab,ti,kw OR 'ligament surger*':ab,ti,kw OR 'ligament reconstruction':ab,ti,kw OR 'spin* surger*':ab,ti,kw OR 'spin* operation*':ab,ti,kw OR 'spin* procedure*':ab,ti,kw OR 'vertebra* surger*':ab,ti,kw OR 'vertebra* operation*':ab,ti,kw OR 'vertebra* procedure*':ab,ti,kw OR 'hip surger*':ab,ti,kw OR 'hip operation*':ab,ti,kw OR 'hip procedure*':ab,ti,kw OR 'hip replacement*':ab,ti,kw	3,907
6	#4 OR #5	16,608
7	'opiate'/exp	5,230
8	opiate*:ab,ti,kw OR opioid*:ab,ti,kw OR 'opioid analgesi*':ab,ti,kw OR oxycodone:ab,ti,kw OR fentanyl:ab,ti,kw OR hydromorphone:ab,ti,kw OR morphine:ab,ti,kw OR oxymorphone:ab,ti,kw OR tramadol:ab,ti,kw	13,972
9	#7 OR #8	14,652
10	'ketamine'/exp	2,103
11	ketamine:ab,ti,kw OR ketalar:ab,ti,kw OR ketaset:ab,ti,kw OR ketanest:ab,ti,kw OR ketajet:ab,ti,kw OR ketavet:ab,ti,kw OR vetamine:ab,ti,kw OR vetaket:ab,ti,kw OR 'ketamine hydrochloride':ab,ti,kw	1,827
12	#10 OR #11	2,243
13	#3 AND #6 AND #9 AND #12	59

**Table 2:** Ovid MEDLINE search strategy.

Search number	Query	Results
1	exp Pain, Postoperative/	6,448
2	('postoperative analgesi*' or 'post-operative analgesi*' or 'post-surg* analgesi*' or 'postsurg* analgesi*' or 'postoperative pain management' or 'post-operative pain management' or 'post-surg* pain management' or 'postsurg* pain management').ab,ti,kf.	1,903
3	1 or 2	6,715
4	exp Orthopedic Procedures/	7,484
5	('orthopedic surger*' or 'orthopedic operation*' or 'orthopedic procedure*' or 'orthopaedic surger*' or 'orthopaedic operation*' or 'orthopaedic procedure*' or 'foot surger*' or 'foot operation*' or 'foot procedure*' or 'podiatric surger*' or 'podiatric operation*' or 'podiatric procedure*' or 'hand surger*' or 'hand operation*' or 'hand procedure*' or 'joint surger*' or 'joint operation*' or 'joint procedure*' or 'joint repair*' or 'ligament surger*' or 'ligament reconstruction' or 'spin* surger*' or 'spin* operation*' or 'spin* procedure*' or 'vertebra* surger*' or 'vertebra* operation*' or 'vertebra* procedure*' or 'hip surger*' or 'hip operation*' or 'hip procedure*' or 'hip replacement*').ab,ti,kf.	1,738
6	4 or 5	8,123
7	exp Analgesics, Opioid/	5,509
8	(opiate* or opioid* or 'opioid analgesi*' or oxycodone or fentanyl or hydromorphone or morphine or oxymorphone or tramadol).ab,ti,kf.	6,555
9	7 or 8	7,530
10	exp Ketamine/	863
11	(ketamine or ketalar or ketaset or ketanest or ketajet or ketavet or vetamine or vetaket or 'ketamine hydrochloride').ab,ti,kf.	865
12	10 or 11	955
13	3 and 6 and 9 and 12	30

**Table 3:** Web of Science search strategy.

Search number	Query	Results
1	TS=(“postoperative analgesi*” or “post-operative analgesi*” or “post-surg* analgesi*” or “postsurg* analgesi*” or “postoperative pain management” or “post-operative pain management” or “post-surg* pain management” or “postsurg* pain management”)	16,801
2	TS=(“orthopedic surger*” or “orthopedic operation*” or “orthopedic procedure*” or “orthopaedic surger*” or “orthopaedic operation*” or “orthopaedic procedure*” or “foot surger*” or “foot operation*” or “foot procedure*” or “podiatric surger*” or “podiatric operation*” or “podiatric procedure*” or “hand surger*” or “hand operation*” or “hand procedure*” or “joint surger*'”or “joint operation*” or “joint procedure*” or “joint repair*” or “ligament surger*” or “ligament reconstruction” or “spin* surger*” or “spin* operation*” or “spin* procedure*” or “vertebra* surger*” or “vertebra* operation*” or “vertebra* procedure*” or “hip surger*” or “hip operation*” or “hip procedure*” or “hip replacement*”)	91,182
3	TS=(opiate* or opioid* or “opioid analgesi*” or oxycodone or fentanyl or hydromorphone or morphine or oxymorphone or tramadol)	225,596
4	TS=(ketamine or ketalar or ketaset or ketanest or ketajet or ketavet or vetamine or vetaket or “ketamine hydrochloride”)	30,198
5	#1 AND #2 AND #3 AND #4	27

**Table 4:** The Cochrane Central Register of Controlled Trials (CENTRAL) in The Cochrane Library search strategy.

Search number	Query	Results
1	MeSH descriptor: [Pain, Postoperative] explode all trees	21,870
2	((postoperative NEXT analgesi*) or (post-operative NEXT analgesi*) or (post-surg* NEXT analgesi*) or (postsurg* NEXT analgesi*) or (postoperative NEXT pain NEXT management) or (post-operative NEXT pain NEXT management) or (post-surg* NEXT pain NEXT management) or (postsurg* NEXT pain NEXT management)):ti,ab,kw	11,552
3	#1 OR #2	30,462
4	MeSH descriptor: [Orthopedic Procedures] explode all trees	20,131
5	(((orthopedic NEXT surger*) or (orthopedic NEXT operation*) or (orthopedic NEXT procedure*) or (orthopaedic NEXT surger*) or (orthopaedic NEXT operation*) or (orthopaedic NEXT procedure*) or (foot NEXT surger*) or (foot NEXT operation*) or (foot NEXT procedure*) or (podiatric NEXT surger*) or (podiatric NEXT operation*) or (podiatric NEXT procedure*) or (hand NEXT surger*) or (hand NEXT operation*) or (hand NEXT procedure*) or (joint NEXT surger*') or (joint NEXT operation*) or (joint NEXT procedure*) or (joint NEXT repair*) or (ligament NEXT surger*) or (ligament NEXT reconstruction) or (spin* NEXT surger*) or (spin* NEXT operation*) or (spin* NEXT procedure*) or (vertebra* NEXT surger*) or (vertebra* NEXT operation*) or (vertebra* NEXT procedure*) or (hip NEXT surger*) or (hip NEXT operation*) or (hip NEXT procedure*) or (hip NEXT replacement*))):ti,ab,kw	12,951
6	#4 OR #5	29,333
7	MeSH descriptor: [Analgesics, Opioid] explode all trees	10,848
8	((opiate* or opioid* or (opioid NEXT analgesi*) or oxycodone or fentanyl or hydromorphone or morphine or oxymorphone or tramadol)):ti,ab,kw	34,039
9	#7 OR #8	39,723
10	MeSH descriptor: [Ketamine] explode all trees	3,147
11	((ketamine or ketalar or ketaset or ketanest or ketajet or ketavet or vetamine or vetaket or (ketamine NEXT hydrochloride))):ti,ab,kw	5,067
12	#10 OR #11	6,426
13	#3 AND #6 AND #9 AND #12	106

**Figure 1:**
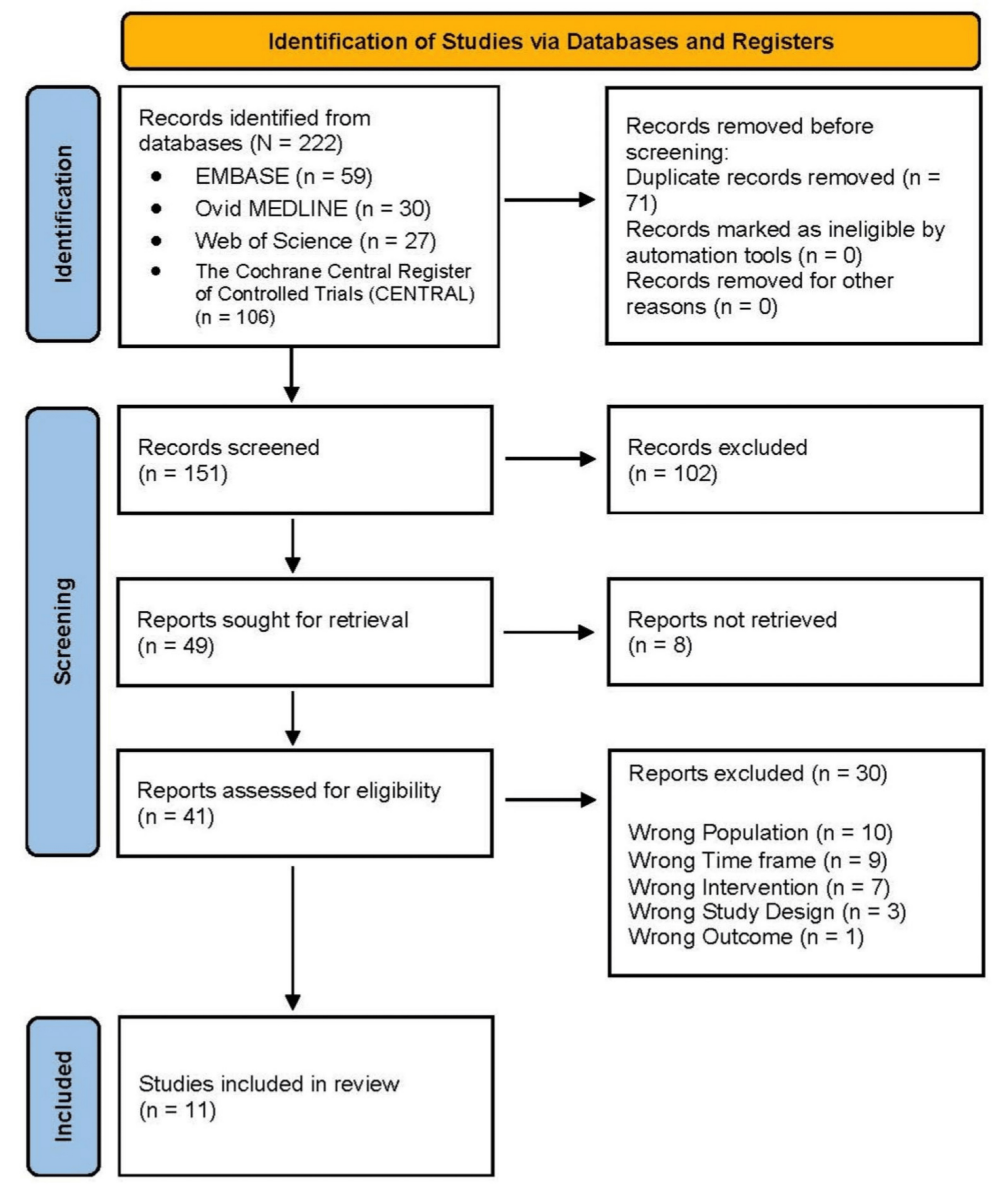
PRISMA screening and selection process. PRISMA: Preferred Reporting Items for Systematic Reviews and Meta-Analysis.

The words contained in the titles, abstracts, and keywords of relevant articles were used to conduct the full search. Only studies published in the English language were included due to the researcher's language limitations. The databases yielded 222 articles, of which 71 were duplicates, leaving 151 records for screening. Two researchers independently screened the titles and abstracts using criteria based on the Population, Intervention, Comparison, and Outcome (PICO) framework to classify studies as “included” or “excluded.” This process resulted in the exclusion of 102 records, leaving 49 articles for full-text review. Of these, eight reports could not be retrieved, resulting in 41 full-text articles assessed for eligibility. Following a detailed review, 30 studies were excluded for the following reasons: wrong population (n = 10), wrong time frame (n = 9), wrong intervention (n = 7), wrong study design (n = 3), and wrong outcome (n = 1). Ultimately, 11 studies met the inclusion criteria. Figure 1 illustrates the study selection process.

Data Extraction

A standardized data extraction form was developed to collect study characteristics, including year, country, study design, sample size, intervention details, outcomes, adverse events, and limitations. Two reviewers independently extracted data, with disagreements resolved by consensus or third-party adjudication. Because of the limited number of included studies and substantial clinical heterogeneity in ketamine dosing, route and timing of administration, orthopedic procedures, and outcome reporting, formal subgroup analyses were not feasible. However, qualitative comparisons based on ketamine dose, route of administration, and timing of administration were incorporated into the narrative synthesis to explore potential sources of variability in treatment effects.

Critical Appraisal of Evidence

The included studies (N = 11) were assessed as having a low risk of bias based on the Joanna Briggs Institute (JBI) critical appraisal tools [20]. Most randomized controlled trials demonstrated appropriate randomization methods and allocation concealment, although reporting of these methods was inconsistent across studies. Blinding of participants and investigators was generally adequate but not uniformly described, introducing potential performance and detection bias in some cases. Variability in outcome measurement, including differences in pain scales and timing of assessment, introduced potential measurement bias. Selective reporting could not be fully excluded, as not all studies reported all predefined outcomes. Despite these limitations, the overall methodological quality of the included studies was acceptable, supporting the validity of the findings while highlighting the need for more standardized reporting in future research. Table 5 summarizes the study-level risk-of-bias assessment for each included trial and demonstrates that overall methodological quality was acceptable, with most concerns related to incomplete reporting of allocation concealment and variability in outcome assessment.

**Table 5:** Study-level summary is presented of methodological quality and risk of bias assessment using the Joanna Briggs Institute (JBI) Critical Appraisal Checklist for Randomized Controlled Trials. Overall, the included studies demonstrated acceptable methodological quality. The most common potential sources of bias included incomplete reporting of study limitations, variability in pain outcome assessment and timing, and incomplete long-term follow-up in one study. These factors were considered during interpretation of the findings but were not judged to substantially compromise the overall validity of the included evidence.

Study	Randomization and blinding	Principal methodological limitation(s)	Overall assessment
Brinck et al. 2021 (IV S-ketamine) [21]	Appropriate randomization and blinding reported	Baseline group imbalances and non-normal data distribution reduced statistical power	Acceptable methodological quality
Brinck et al. 2021 (S-ketamine PCA) [22]	Appropriate randomization and blinding reported	Reduction in opioid consumption not accompanied by reduced opioid-related adverse events	Acceptable methodological quality
Cengiz et al. 2014 [23]	Appropriate randomization and blinding reported	Pain outcomes assessed only during the first 24 postoperative hours	Acceptable methodological quality
Giri et al. 2018 [24]	Randomized and blinded study	Study limitations not reported by authors	Acceptable methodological quality
Jelodar et al. 2023 [25]	Randomized and blinded study	Study limitations not reported by authors	Acceptable methodological quality
Khashan et al. 2015 [26]	Appropriate randomization and blinding reported	Possible intra-articular drug washout during arthroscopy	Acceptable methodological quality
Martinez et al. 2014 [27]	Appropriate randomization and blinding reported	Study limitations not reported by authors	Acceptable methodological quality
Nielsen et al. 2017 [28]	Randomized, blinded controlled trial	Approximately 65% long-term follow-up; variability in pain assessment methods	Acceptable methodological quality
Paulin et al. 2021 [29]	Appropriate randomization and blinding reported	Ketamine limited to intraoperative administration; inflammatory markers not assessed	Acceptable methodological quality
Prabhakar et al. 2024 [30]	Randomized controlled trial	Single-center study; limited dosing evaluation; no systemic opioid comparator	Acceptable methodological quality
Sharma et al. 2024 [31]	Appropriate randomization and blinding reported	Study limitations not reported by authors	Acceptable methodological quality

Results

Characteristics of the Studies

To facilitate comparisons across studies, the included studies were grouped in a table organized by study design, year of publication, methods, and purpose. The articles were then analyzed for outcomes and key findings, adverse events, and limitations. Eleven studies met the inclusion criteria, including predominantly randomized controlled trials along with controlled clinical trials.

The included studies consisted primarily of randomized controlled trials evaluating perioperative ketamine use in adult patients undergoing various orthopedic procedures, including spinal fusion, total knee arthroplasty, total hip arthroplasty, and arthroscopic repairs [21-31]. Variability was observed across studies in ketamine dosing, timing, and route of administration, as well as in outcome reporting.

Findings are presented across three domains: postoperative opioid consumption, pain intensity, and adverse events. Of the 11 included studies, five demonstrated statistically significant reductions in postoperative opioid consumption with adjunctive ketamine, while four reported statistically significant improvements in patient-reported pain scores. Across all studies, no significant increase in adverse events was observed, with several reporting reductions in opioid-related side effects.

Postoperative Opioid Consumption

Across the included studies, perioperative ketamine administration was frequently associated with reduced postoperative opioid consumption. Many studies demonstrated a statistically significant reduction in opioid requirements in patients receiving ketamine compared to opioid-only regimens.

Several randomized controlled trials reported notable reductions in opioid use within the first 24 hours postoperatively. Cengiz et al. demonstrated significantly lower cumulative morphine consumption in the ketamine group compared to placebo (47.0 mg versus 85.2 mg, p<0.001) [23]. Comparable findings were observed across multiple studies, demonstrating reductions in opioid requirements across different orthopedic procedures and postoperative time points [22-25,27,28,31]. Nielsen et al. reported reduced 24-hour morphine consumption following lumbar fusion surgery in patients receiving ketamine compared to the placebo group (79 vs 121 mg, p<0.001).

One study also demonstrated a statistically significant reduction in non-opioid analgesic requirements (diclofenac) and delayed time to first analgesic request, although this did not specifically reflect opioid consumption [24].

The extent of reduction varied across studies but demonstrated a frequent trend favoring ketamine use. Intravenous administration, particularly when initiated at induction and continued intraoperatively, was associated with the most pronounced reductions in opioid consumption. Across studies demonstrating benefit, postoperative opioid consumption was reduced by approximately 25% to 45% during the first 24 postoperative hours, although the magnitude varied according to ketamine dose, route of administration, surgical procedure, and comparator regimen. For example, Cengiz et al. reported a 45% reduction in cumulative morphine consumption (47.0 mg vs 85.2 mg), while Nielsen et al. reported an approximately 35% reduction following lumbar fusion surgery (79 mg vs 121 mg).

Patient-Reported Pain Intensity

Patient-reported pain outcomes, assessed using the NRS and VAS [18,19], were heterogeneous across studies. Three studies reported statistically significant reductions in postoperative pain scores with ketamine use. Cengiz et al. demonstrated significantly lower VAS scores at 24 hours in the ketamine group compared to placebo (2 vs 6.3 mm, p<0.001) [23]. Sharma et al. observed significantly lower NRS scores in patients receiving ketamine, particularly when administered preemptively at induction [31]. One study reported a statistically significant reduction in pain scores in the highest-dose ketamine group, suggesting a dose-dependent effect [22]. However, other studies reported no significant differences in pain scores between ketamine and control groups despite reductions in opioid consumption [21,26-28,30]. Paulin [29] also found no significant difference, but that study focused more broadly on recovery and satisfaction rather than just pain, so leaving it out is reasonable. Jelodar et al. demonstrated statistically significant improvements in postoperative pain scores with both ketamine and dexmedetomidine as adjuncts to morphine compared to morphine alone; however, dexmedetomidine was associated with greater pain reduction than ketamine [25].

Overall, while ketamine as an adjunct suggested a potential improvement in pain control, findings were inconsistent, with only a subset of studies demonstrating statistically significant improvements. Reductions in opioid consumption were not uniformly accompanied by significant improvements in patient-reported pain intensity.

Adverse Events

Across the included studies, perioperative ketamine demonstrated a safety profile comparable to opioid-only regimens. No consistent increase in adverse events was observed with ketamine use. Common postoperative adverse events, including nausea, hallucinations, and nightmares, were reported at similar rates between ketamine and control groups, with no statistically significant differences in most studies. Notably, concerns regarding ketamine-associated psychomimetic effects were not substantiated by consistent findings across the included literature.

Three studies reported reductions in opioid-related adverse effects in the ketamine groups. For example, decreased incidence of pruritus and lower levels of postoperative sedation were observed in select studies [24,27]. However, these findings were not consistently replicated across all studies. Table 6 reports on the characteristics of the included studies.

**Table 6:** This table summarizes the characteristics of the 11 included studies evaluating perioperative ketamine as an adjunct to opioid therapy for postoperative pain management in adult orthopedic surgical populations. Data presented include study design, sample size (N), intervention details (including ketamine dosing, timing, and route of administration), key findings related to postoperative opioid consumption and patient-reported pain outcomes, reported adverse events, and study limitations. IA: intra-articular; IV: intravenous; NRS: Numeric Rating Scale; PCA: patient-controlled analgesia; PO: oral; VAS: Visual Analog Scale.

Author and year	Country	Purpose	Study design	Methods	Key findings	Adverse events	Limitations
Brinck et al. 2021 (IV S-ketamine) [21]	Finland	To evaluate dose-dependent intraoperative S-ketamine in lumbar fusion surgery	Double-blind randomized controlled trial; n = 198 (three groups)	S-ketamine bolus (0.5 mg/kg) + infusion (0.12 or 0.6 mg/kg/h) vs placebo	No statistically significant reduction in 48-hour opioid consumption vs placebo; transient early postoperative pain reduction observed (not statistically significant); higher dose associated with increased sedation.	Adverse events largely comparable; increased sedation with a higher dose	Non-normal data distribution reduced statistical power; imbalances in group baseline variables
Brinck et al. 2021 (S-ketamine PCA) [22]	Finland	To assess dose-dependent effects of S-ketamine added to oxycodone PCA on postoperative opioid consumption.	Double-blind randomized controlled trial; n = 100 (four groups, n = 25 each)	PCA oxycodone (1 mg/mL) with or without S-ketamine (ratios 1:0.25, 1:0.5, 1:0.75)	S-ketamine at a 1:0.75 ratio significantly reduced 24-hour oxycodone consumption compared to control and significantly reduced pain scores during the early postoperative period (no sustained differences in pain scores at later time points).	Incidence of nausea, pruritus, and nightmares comparable across groups	Reduction in opioid consumption did not correspond to reduced opioid-related adverse events
Cengiz et al. 2014 [23]	Turkey	To assess intraoperative ketamine infusion on postoperative pain following total knee replacement	Double-blind randomized controlled trial; n = 60 (ketamine n = 30, placebo n = 30)	Ketamine infusion (6 µg/kg/min) intraoperatively vs saline	The ketamine group had significantly lower postoperative pain scores and significantly reduced morphine consumption vs placebo; longer time to first analgesic request.	Incidence of adverse events comparable between groups	Pain outcomes only assessed during first 24 hours
Giri et al. 2018 [24]	India	To compare ketamine vs nalbuphine applied epidurally via gelfoam in spinal surgery	Double-blind randomized controlled trial; n = 60 (three groups, n = 20 each)	Gelfoam soaked with ketamine, nalbuphine, or saline placed epidurally	Significantly reduced rescue analgesic consumption (diclofenac) and delayed time to first analgesic request vs nalbuphine; lower pain scores observed (statistical significance not clearly reported).	Higher sedation observed in ketamine group	Limitations not reported
Jelodar et al. 2023 [25]	Iran	To compare ketamine and dexmedetomidine as adjuvants to morphine PCA	Double-blind randomized controlled trial; n = 196 (three groups)	Morphine PCA with ketamine, dexmedetomidine, or placebo	Significantly improved postoperative pain scores with ketamine + morphine vs morphine alone; the dexmedetomidine group demonstrated greater pain reduction compared to ketamine.	Not reported	Limitations not reported
Khashan et al. 2015 [26]	Israel	To evaluate intra-articular ketamine combined with morphine for postoperative analgesia	Double-blind randomized controlled trial; n = 45 (three groups, n = 15 each)	IA morphine, IA ketamine + morphine, or saline	No statistically significant difference in pain scores or opioid consumption vs morphine alone.	Not reported	Potential washout of intra-articular drugs during arthroscopy; drug leakage in large rotator cuff tears
Martinez et al. 2014 [27]	France	To evaluate ketamine and pregabalin, alone and combined, on postoperative pain and opioid consumption	Double-blind randomized controlled trial; n = 120 (four groups)	Ketamine (0.5 mg/kg bolus + infusion), pregabalin (150 mg PO), combination, or placebo	Ketamine and pregabalin each significantly reduced morphine consumption vs placebo; the combination produced the greatest reduction. No statistically significant difference in pain scores.	Adverse events comparable across groups	Limitations not reported
Nielsen et al. 2017 [28]	Denmark	To evaluate intraoperative ketamine in opioid-dependent patients undergoing spinal fusion	Randomized, blinded controlled trial; n = 147 (ketamine n = 74, placebo n = 73)	Ketamine: 0.5 mg/kg bolus + 0.25 mg/kg/h infusion intraoperatively; placebo saline	Significantly reduced postoperative opioid consumption; no statistically significant difference in pain scores.	No significant differences in nausea, hallucinations, or nightmares; sedation reduced in ketamine group	65% follow-up at six months; inconsistent pain assessment methods
Paulin et al. 2021 [29]	India	To evaluate the effect of intraoperative ketamine on postoperative recovery, opioid consumption, pain, and patient satisfaction	Double-blind randomized controlled trial; n = 52 (ketamine n = 26, saline n = 26)	The ketamine group received a 0.5 mg/kg IV bolus prior to incision followed by intraoperative infusion (10 µg/kg/min). Control received saline.	No statistically significant differences in opioid consumption or pain scores.	No significant adverse events reported	Ketamine limited to intraoperative period; inflammatory markers not assessed
Prabhakar et al. 2024 [30]	India	To evaluate addition of ketamine to epidural morphine for postoperative analgesia	Randomized controlled trial; n = 48 (morphine n = 24, morphine + ketamine n = 24)	Epidural morphine ± ketamine (30 mg)	No statistically significant improvement in pain scores or postoperative outcomes.	Nausea reported; no significant differences between groups	Single-center study; limited dosing evaluation; lack of systemic opioid comparison
Sharma et al. 2024 [31]	India	To compare timing of ketamine administration on postoperative pain and opioid consumption	Double-blind randomized controlled trial; n = 75 (three groups, n = 25 each)	Ketamine given pre-incision, post-closure, or placebo	Significantly lower pain scores and significantly reduced opioid consumption with preemptive ketamine vs comparator groups.	Nausea reported across groups; no major differences noted	Limitations not reported

To facilitate comparison across studies, Table 7 summarizes ketamine administration strategies and their associated clinical outcomes.

**Table 7:** Summary of ketamine administration strategies and clinical outcomes among the included studies is presented. Intravenous ketamine administered before surgical incision and continued intraoperatively demonstrated the most consistent opioid-sparing effects. Epidural and intra-articular administration generally produced less consistent benefits. Improvements in patient-reported pain intensity were less consistent than reductions in postoperative opioid consumption. IA: intra-articular; IV: intravenous; PCA: patient-controlled analgesia.

Study	Route	Timing	Strategy	Approximate opioid reduction	Pain reduction
Brinck et al. 2021 (IV S-ketamine) [21]	IV	Intra-op	Bolus + infusion	No significant reduction	No
Brinck et al. 2021 (S-ketamine PCA) [22]	PCA	Post-op	PCA admixture	Significant (dose-dependent)	Early only
Cengiz et al. 2014 [23]	IV infusion	Intra-op	Continuous infusion	≈45% reduction	Yes
Giri et al. 2018 [24]	Epidural	Intra-op	Gelfoam	Rescue analgesics reduced	Yes
Jelodar et al. 2023 [25]	PCA	Post-op	PCA admixture	Not primary endpoint	Yes
Khashan et al. 2015 [26]	IA	End of surgery	IA ketamine	No significant reduction	No
Martinez et al. 2014 [27]	IV	Pre-incision + intra-op	Bolus + infusion	Significant reduction	No
Nielsen et al. 2017 [28]	IV	Intra-op	Bolus + infusion	≈35% reduction	No
Paulin et al. 2021 [29]	IV	Pre-incision + intra-op	Bolus + infusion	No significant reduction	No
Prabhakar et al. 2024 [30]	Epidural	Intra-op	Epidural ketamine	No significant reduction	No
Sharma et al. 2024 [31]	IV	Pre-incision vs. post-closure	Timing study	Significant reduction	Yes

Discussion

The most consistent finding across the included studies was a reduction in postoperative opioid requirements, particularly during the first 24 hours following surgery [22-24,27,28,31]. Although the magnitude of benefit varied, these findings suggest that perioperative ketamine may serve as an effective opioid-sparing adjunct across a range of orthopedic procedures. Studies employing intravenous ketamine initiated before surgical incision and continued intraoperatively generally demonstrated the most favorable outcomes [23,27-29,31], consistent with ketamine's antagonism of the N-methyl-D-aspartate (NMDA) receptor and its ability to attenuate central sensitization [9].

In contrast, improvements in patient-reported pain intensity were less consistent. Although several studies demonstrated statistically significant reductions in postoperative pain scores [22,23,25,31], others found no significant differences despite reductions in opioid consumption [21,26-28,30]. This discordance likely reflects heterogeneity in study design, ketamine dosing regimens, timing and route of administration, and pain assessment methods, including whether pain was assessed at rest or during movement and during the early or later postoperative period. These differences in outcome assessment may have contributed to the variability in reported analgesic efficacy across studies. It also suggests that reduced opioid consumption does not necessarily translate into measurable improvements in pain scores, particularly when baseline multimodal analgesia already provides adequate pain control.

Route and timing of ketamine administration also appeared to influence treatment response. Intravenous administration initiated before incision and continued throughout surgery produced the most consistent opioid-sparing effects and, in some studies, improved postoperative analgesia [23,27-29,31]. In contrast, studies evaluating epidural, intra-articular, or postoperative ketamine administration generally reported less consistent clinical benefit [24,26,30]. These findings further support the concept that ketamine is most effective when administered as a preemptive analgesic to reduce central sensitization before and during surgical tissue injury [9]. However, this observation represents an overall qualitative interpretation of the included studies rather than a predefined subgroup analysis and should therefore be interpreted cautiously.

Across the included studies, perioperative ketamine demonstrated a favorable safety profile. No consistent increase in adverse events was observed compared with opioid-only regimens [11,22,27,28]. Furthermore, several investigations reported reductions in opioid-related adverse effects, including pruritus and postoperative sedation [22,24,27,28], suggesting that ketamine's opioid-sparing effects may provide indirect safety benefits. Although isolated reports described increased sedation with higher ketamine doses, these findings were not consistently replicated.

Collectively, these findings support the incorporation of ketamine into multimodal analgesia protocols designed to reduce perioperative opioid exposure [8,10-12]. Such an approach may be particularly valuable for patients at increased risk for opioid-related complications or prolonged postoperative opioid use. However, because improvements in pain intensity were less consistent than reductions in opioid consumption, ketamine should be viewed primarily as an opioid-sparing adjunct rather than as a replacement for conventional postoperative analgesic strategies.

Limitations

This review is limited by significant heterogeneity across studies in ketamine dosing, timing, route of administration, and outcome reporting, which restricted direct comparison and precluded meta-analysis. The relatively small number of studies included and variation in study design may limit generalizability. Additionally, restriction to English-language publications introduces potential selection bias. Furthermore, because gray literature was not searched, publication bias cannot be excluded, as unpublished studies with neutral or negative findings may not have been identified. Variability in pain assessment methods and timepoints further complicates interpretation of analgesic outcomes. Because only randomized and controlled clinical trials were included, potentially informative observational studies were excluded.

The findings of this review are generally consistent with previous systematic reviews and meta-analyses demonstrating that perioperative ketamine provides a reliable opioid-sparing effect while producing less consistent improvements in postoperative pain intensity [10,11]. By focusing on contemporary randomized controlled trials in orthopedic surgery, the present review further highlights how ketamine dose, route, and timing of administration may contribute to variability in clinical outcomes.

Future Research

Future research should focus on standardizing ketamine administration protocols, including optimal dosing, timing, and route of delivery. Future investigations should also examine patient-centered outcomes, including functional recovery, quality of recovery, length of hospital stay, and persistent postoperative opioid use. Comparative studies evaluating different ketamine formulations and administration strategies are needed to clarify its role in perioperative care. Additionally, longitudinal studies assessing subacute and chronic pain outcomes would help define the broader clinical impact of perioperative ketamine use.

## Conclusions

Perioperative ketamine appears to be an effective opioid-sparing adjunct in adult patients undergoing orthopedic surgery, particularly during the immediate postoperative period. Across the included studies, ketamine was frequently associated with reduced postoperative opioid consumption without a corresponding increase in adverse events, although improvements in patient-reported pain intensity were less consistent. The reduction in opioid exposure alone may provide meaningful clinical benefit, especially in patients at increased risk for opioid-related complications or prolonged postoperative opioid use. These findings support the incorporation of ketamine into multimodal analgesia protocols as part of broader opioid stewardship efforts in perioperative care. Intravenous ketamine administered preemptively and continued intraoperatively demonstrated the most consistent benefit across studies; however, substantial heterogeneity in dosing strategies, timing of administration, routes of delivery, and outcome reporting limits the ability to establish standardized clinical recommendations. Future high-quality randomized controlled trials using standardized pain assessment measures and ketamine protocols are needed to clarify optimal dosing strategies, determine long-term outcomes, and better define which orthopedic patient populations are most likely to derive the greatest benefit from perioperative ketamine therapy.
